# Evaluating Two Fungicides, Prochloraz–Manganese Chloride Complex and Seboctylamine Acetate, to Control Cobweb Disease in White Button Mushroom Caused by *Cladobotryum mycophilum*

**DOI:** 10.3390/jof10100676

**Published:** 2024-09-27

**Authors:** Qiqi Chen, Yazhen Yuan, Gang Chen, Ning Li, Xinrong Li, Yufei Lan, Hongyan Wang

**Affiliations:** 1 Department of Plant Protection, Shandong Agricultural University, Tai’an 271018, China; qiqichen19970905@163.com (Q.C.); cg15052715639@163.com (G.C.); 13465333875@163.com (N.L.); lxr15550892735@163.com (X.L.); 2 Tai’an Academy of Agricultural Sciences, Tai’an 271018, China; lanyufei526@163.com

**Keywords:** *Agaricus bisporus*, *Cladobotryum mycophilum*, cobweb disease, seboctylamine acetate, prochloraz–manganese chloride complex

## Abstract

Cobweb disease in white button mushroom (*Agaricus bisporus*) is a newly identified disease caused by *Cladobotryum mycophilum* in China. Currently, there are few highly effective and safe fungicides for controlling this disease in the field. This study assessed the fungicidal effect of prochloraz–manganese chloride complex and seboctylamine acetate against *C. mycophilum*, as well as their ability to control cobweb disease. Additionally, the residues of these fungicides in the mycelium and the mushroom were evaluated. The extent of the fungicidal effect against the pathogen was determined based on the efficiency of crop production. The results revealed that, in addition to the potent inhibitory effect of prochloraz–manganese chloride complex on the hyphae of *C. mycophilum*, the domestically developed seboctylamine acetate exhibited high toxicity, inhibiting both mycelial growth and spore germination of *C. mycophilum*, with EC_50_ values of 0.990 mg/L and 0.652 mg/L, respectively. Furthermore, the application of the two chemical agents had no adverse effects on the mycelial growth and fruiting body growth of *A. bisporus*, and the residual amount of chemical agent was lower than the maximum residue limit standard. The field application results showed that 400 mg/L of prochloraz–manganese chloride complex and 6 mg/L of seboctylamine acetate resulted in 61.38% and 81.17% disease control respectively. This study presents efficient and safe fungicides for controlling cobweb disease in white button mushroom. Additionally, a residue determination analysis of the fungicide seboctylamine acetate in mushroom crops is described.

## 1. Introduction

Edible fungi are important in the diets of people all over the world [1]. As the edible fungus industry has flourished and expanded its scale significantly [2], China has become a major producer in this sector [3]. The species *Agaricus bisporus* (*A. bisporus*), also known as the white button mushroom [4], is widely cultivated, has a wide distribution range and high production yield, and is highly favored [5,6]. The *A. bisporus* mushroom has tender flesh, a delicious taste and excellent commodity value due to its high protein content, low fat and low calories [7]. On the other hand, its medicinal value is also very high, and it has a certain preventive effect on viral diseases, stomach diseases, etc. [8].

With the rapid development of the *A. bisporus* planting industry, the occurrence of *A. bisporus* diseases is becoming more common [9], such as ulcer disease, brown spot, soft rot, and cobweb disease. The yield is generally reduced by 20~30% when these diseases occur [10]. Cobweb disease is particularly severely affecting *A. bisporus* in China [11], and is caused by *Cladobotryum mycophilum* (*C. mycophilum*). It can infect various edible fungi such as *Lentinula edodes* [11]. Cobweb disease, together with *Lecanicillium fungicola*, *Trichoderma aggressivum* and *Mycogone perniciosa*, are acknowledged as the four primary diseases of edible fungi attributed to parasitic fungi [5]. In the initial stages of *A. bisporus* cobweb disease, downy mildew-like mycelium adheres to the soil layer and gradually spreads, eventually reaching the tip of the fruiting body [12]. In one study, small white round patches appeared in the casing soil or basidiospore. As the mycelium of the pathogen progressed, it gradually took on a cottony, flocculent appearance and simultaneously produced a copious number of dry spores. These spores were highly susceptible to being dislodged upon physical disturbance, such as watering, whereupon the conidia were disseminated throughout the mushroom facility via air currents, ultimately causing the appearance of brown spots on the fruiting bodies of infected *A. bisporus* mushrooms [13]. Finally, they rotted, and the whole fruiting body shrunk if it was serious. The pathogen can infect *A. bisporus* throughout the entire development period [14]. To prevent the spread of cobweb disease, it is crucial to take immediate action before spatulation sets in. This can be achieved by covering the affected area with a thick, dampened paper sheet to hinder the release of conidia and arrest the further dissemination of the disease [15].

Prevention and control strategies for edible mushroom diseases encompass the breeding of disease-resistant varieties, rigorous strain quality control, meticulous cultivation environment management, the induction and application of disease resistance mechanisms, chemical intervention, biological control methods, and more [16]. However, the breeding of excellent strains takes a long time and is prone to variation [15]. While biological pesticides are indeed safe for humans and the environment, there remains a lack of an effective biological control method specifically tailored to combatting cobweb disease. Furthermore, the exact mechanism of action of these potential biological agents is not yet fully understood. Consequently, in field applications, their control efficacy often falls short of that achieved by chemical fungicides [17]. Therefore, the control of mushroom diseases still depends on the use of chemical fungicides [18]. In China, only seven fungicides, including prochloraz–manganese chloride complex, are allowed to be used on mushrooms, but there is no registered fungicide for the prevention and control of cobweb disease on *A. bisporus*. Prochloraz–manganese salt, also known as prochloraz–manganese chloride complex, is composed of prochloraz and manganese chloride. Its disease prevention performance is very similar to prochloraz. Prochloraz–manganese was found to control green mold on white mushrooms (*A. bisporus*) and increase the yield of *A. bisporus*, and can effectively control wet bubble disease (WBD) caused by *Mycogone rosea* [19]. Chakwiya et al. focused on the sensitivity of *C. mycophilum* to carbendazim and prochloraz–manganese; the prochloraz–manganese ED_50_ values varied from 0.00001 mg/L to 0.55 mg/L, and it is to be used in a disease management strategy [20]. Pathogen sensitivity to fungicides was estimated by probit analyses. Fungicide susceptibility tests showed that *C. mycophilum* strains were highly sensitive both to prochloraz (ED_50_ < 0.087 μg mL^−1^) and the newly introduced metrafenone (ED_50_ < 0.15 μg mL^−1^) [21]. Prochloraz can no longer be used in the European Union (EU) as of June 2023, and over-reliance on metrafenone has resulted in putative resistant pathogenic strains emerging. Prochloraz still showed good control of two different isolates of *C. mycophilum*, with efficacy values consistently reaching 70% [22]. However, some studies found that *C. mycophilum* type II isolate 192B1 was sensitive to prochloraz-Mn, and easily lead to drug resistance, resulting in its inability to prevent the occurrence of cobweb disease symptoms [23]. Consequently, there is an urgent need to identify chemical agents capable of effectively managing this disease.

In recent years, extensive research has highlighted that the presence of chemicals in the human body, even at trace levels, poses a significant risk to health. Therefore, the stringent regulation of pesticide residue levels in agricultural products, along with regular comparison to the Maximum Residue Limits (MRLs), and a concerted effort to promote reduced pesticide usage, are crucial steps towards ensuring the safety of our food supply [24]. Various methods have been developed for detecting residual pesticides over time, including the ultrasound-assisted solvent extraction, solid phase extraction and QuEChERS methods. Analytical techniques such as high-performance liquid chromatography (HPLC) and high-performance liquid chromatography–mass spectrometry (HPLC-MS) have been widely applied in this context [25]. The advancement and implementation of the aforementioned pesticide residue detection technologies have significantly enhanced analysis accuracy, streamlined procedural complexity, and bolstered analysis efficiency [26]. Among these methods, HPLC-MS is notable for its high resolution and ability to perform both qualitative and quantitative analysis [27].

**Figure 1:**
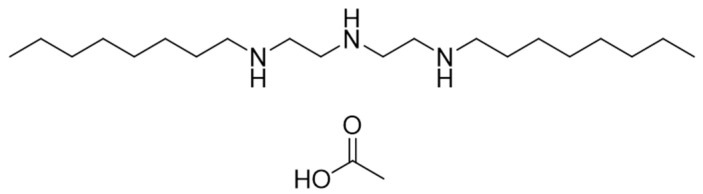
The structure of seboctylamine acetate.

In this study, we conducted an indoor toxicity test to evaluate the antifungal effects of prochloraz–manganese chloride complex and seboctylamine acetate (former name: Xinjuan; the chemical structure is shown in Figure 1), which were selected as the agents with a higher antifungal effect than *A. bisporus* cobweb disease. The control effects of prochloraz–manganese chloride complex and seboctylamine acetate were investigated for their effectiveness against the cobweb disease pathogen in the field. Additionally, the safety of these compounds on the mycelial growth and fruiting body development of *A. bisporus* was assessed. Meanwhile, the method for detecting the residue of seboctylamine acetate in *A. bisporus* was established, and the effect of the level of chemical on yield was thoroughly explored, to provide a basis for the development of application rates and the risk assessment of fungicides in the cultivation of *A. bisporus*.

## 2. Materials and Methods

### 2.1. Test Strains and Fungicides

The first step was to investigate the strain *C. mycophilum* at the Tai’an Academy of Agricultural Science in Shandong Province. The researchers collected and isolated the fruiting bodies of *A. bisporus* cobweb disease from a mushroom shed in Jining, and purified and identified the strain as *C. mycophilum*. They stored it at 4 °C for later use. 

*A. bisporus* varieties S2796, W192, and W2000 were provided by Tai’an Academy of Agricultural Sciences. 

Several fungicides, including 97% quatrimycin TC, purchased by Shandong Union Pesticide Industry Co., Ltd. in Taian, China; 98% pyrimethanil TC, purchased by Shandong Weifang Rainbow Chemical Co., Ltd. in Weifang, China; 97% kresoxim-methyl TC, purchased by Shandong Hailier Chemical Co., Ltd. in Qingdao, China; 97% difenoconazole TC, purchased by Shandong Union Pesticide Industry Co., Ltd. in Tai’an, China; 96% tebuconazole TC, purchased by Shandong Union Pesticide Industry Co., Ltd. in Tai’an, China; 98% prochloraz–manganese chloride complex TC, purchased by in China Shandong Weifang Rainbow Chemical Co., Ltd. in Weifang, China; 97% prothioconazole TC, purchased by Shandong Hailier Chemical Co., Ltd. in Qingdao, China; 34% polyoxin TC, purchased by Shandong Yucheng Biochemical Pesticide Co., Ltd. in Weifang, China; 20% seboctylamine acetate AS, purchased by Weifang Voelsing Biopesticide Co., Ltd. in Weifang, China; 50% prochloraz–manganese chloride complex WP, purchased by FMC Investment Co., Ltd. in Philadelphia, PA, USA; and 1.8% seboctylamine acetate AS, purchased by Shaanxi Xi’an Jiake Agricultural Chemical Co., Ltd. in Xi’an, China.

### 2.2. Toxicity Measurement

To measure toxicity, the indoor biological activity of fungicides against *C. mycophilum* was determined by the mycelial growth rate method [28]. A small amount of acetone was used to dissolve the following fungicides: 97% kresoxim-methyl, 97% quatrimycin, 97% difenoconazole, 96% tebuconazole, 98% prochloraz–manganese chloride complex, 34% polyoxin TC, 97% prothioconazole, and 98% pyrimethanil. The dissolved fungicides were then diluted with sterile water. Additionally, 20% seboctylamine acetate aqueous solution and 50% prochloraz–manganese chloride complex WP were separately dissolved in sterile water to create a 1 × 10^4^ mg/L mother solution. This mother solution was stored in a refrigerator at 4 °C for a brief period.

To prepare the drug-containing medium, 45 mL of pre-prepared PDA medium was added to a 100 mL pre-sterilized and dry Erlenmeyer flask. Following this, 5 mL aliquots of the drug solution were added in ascending order of concentration, with thorough shaking after each addition. The resulting mixture was then poured into three 9 cm diameter Petri dishes to create a drug-containing plate with the desired test design concentration [29]. The treatment without fungicide was set as a blank control, and each treatment had 3 replicates. The final effective component dosage of each agent is shown in Table 1.

The indoor toxicity was assessed using the mycelium growth rate method [30]. The *C. mycophilum* mycelium was collected from the edge of the mycelium using a sterile puncher (5 mm diameter) and inoculated onto a PDA plate containing the drug. The culture dish was placed in a dark environment with a temperature of 25 ± 1 °C for culturing. The growth diameter of the mycelium in each treatment group, along with that of the blank control, was precisely measured when the mycelium of the blank control group had covered three-quarters of the area within the Petri dish. The inhibition rate of the fungicide on mycelial growth was calculated using following formula:

Growth inhibition rate (%) = [(Pathogen diameter in control − Pathogen diameter under fungicidal action)/Pathogen diameter in control] × 100.

The virulence regression equation and EC_50_ value were also calculated. 

### 2.3. Spore Germination Test

In the spore germination test, the indoor biological activity of fungicides against *A. bisporus* cobweb disease was determined using the spore germination method [31]. The final effective component dosage of each agent is shown in Table 2. The spore suspension was prepared using sterile water, and its concentration was adjusted to 10^6^ spores per milliliter. Subsequently, 20 microliters of this spore suspension was pipetted onto a concave glass slide. To prepare the fungicide-containing spore suspension, 20 microliters of each respective concentration gradient of the fungicide was then added to the spore suspension on the slide. The control group was administered an equivalent volume of sterile water. Each treatment was replicated three times to ensure the reliability and reproducibility of the results. The concave slides containing the prepared spore suspensions were carefully placed in Petri dishes and then incubated at a constant temperature of 25 °C in a dark, humid environment. After 24 h of incubation, the spore germination was meticulously examined under a microscope to assess the effectiveness of the treatments. Each treatment was randomly observed in three fields, totaling 100 spores. Spore production was assessed using the blood cell counting plate method, and the spore germination rate and drug inhibition rate during spore germination were calculated after 5 days. The relevant calculation formula is as follows:

Spore germination rate (%) = spore germination number/total number of spores examined × 100%

Spore germination inhibition rate (%) = (spore germination rate of control group − spore germination rate of treatment group)/spore germination rate of control group × 100%

The calculation method of efficiency improvement is the same as above.

### 2.4. Effect of Fungicides on Mycelial Growth of A. bisporus

The indoor effectiveness of fungicides on a *A. bisporus* was determined by the mycelium growth rate method [32]. A 98% prochloraz–manganese chloride complex and 20% seboctylamine acetate aqueous solution were dissolved and diluted with sterile water. The mother liquor concentration was 1 × 10^4^ mg/L, and it was stored in a refrigerator at 4 °C. The test gradients of prochloraz–manganese chloride complex were 0.94 mg/L, 1.88 mg/L, and 3.76 mg/L, while test gradients of seboctylamine acetate were 0.99 mg/L, 1.98 mg/L, and 3.96 mg/L.

The preparation of drug-containing medium was as follows: A total of 45 mL of pre-prepared PDA medium was dispensed into a 100 mL pre-sterilized Erlenmeyer flask, and the mixture was poured into three 9 cm diameter Petri dishes to create drug-containing plates with the desired test concentrations. The concentration range of fungicides affecting the growth of *A. bisporus* mycelium was designed according to the method of Gao et al. (2017), and the medium plates with three treatment concentrations were prepared for use [30]. The treatment without adding chemicals was set as a blank control, and each treatment was repeated 3 times. The *C. mycophilum* mycelium was obtained from the edge of the mycelium using a sterile puncher (5 mm diameter) and inoculated onto a PDA plate containing the drug. The culture dish was placed in a dark environment at a temperature of 25 ± 1 °C. The growth diameter of each treatment and blank control mycelium was measured when the blank control covered three-quarters of the Petri dish. The inhibition rate of each fungicide during mycelial growth was calculated using the following formula [33]:

Growth inhibition rate (%) = [(Pathogen diameter in control − Pathogen diameter under fungicidal action)/Pathogen diameter in control] × 100.

### 2.5. Field Safety of Fungicides on A. bisporus

According to the requirements of NY/T 1965.1-2010 [34], the fungicide test was conducted once, twice and four times the recommended dosage in the field, using the dosage of active ingredients. Spraying treatment was carried out when the mushroom buds rose from the covering soil, and the test results were investigated after 10 days. The specific fungicide dosage was as follows: 6, 12, and 24 mg/L of 20% aqueous seboctylamine acetate solution; the concentrations of the 98% prochloraz–manganese chloride complex TC were set at 400 mg/L, 800 mg/L, and 1600 mg/L. The water treatment without the agent was used as the blank control, and each treatment was sprayed with 250 mL/m^2^ fungicide solution. Each plot was arranged in random blocks, and each treatment was repeated three times. Using the five-point sampling method, 20 fruiting bodies were collected from each cell, and the area of each plot was 2 m^2^.

### 2.6. Field Control Efficacy

The experiment was carried out in the mushroom shed of the mushroom resources and utilization practice base of Shandong Agricultural University in Tai’an City, China, in October 2020, and the experiment was conducted in the mushroom shed for the continuous cultivation of *A. bisporus*. The previous crop was *A. bisporus*, and all the experimental areas conformed to local scientific agricultural practice, and the cultivation conditions were uniform.

The variety of *A. bisporus* was S2796. When the daytime temperature stabilized at 20–24 °C and the culture material remained below 28 °C, the strain was sown. Initially, half the inoculum was spread on the material’s surface, and the remaining inoculum was evenly distributed. After about 20 days, the mycelium was ready for soil covering. The prepared spores of *A. bisporus* cobweb disease were covered with soil 5 days later.

The mushroom buds were sprayed at the beginning of 13 October 2020. The experiment was divided into seven treatments: 50% prochloraz–manganese chloride complex WP 400, 800, and 1600 mg/L; 1.8% seboctylamine acetate AS 2, 4, and 6 mg/L; and a blank control. Each treatment was sprayed with 250 mL/m^2^ fungicide solution, and each plot was arranged randomly.

Based on the NY/T 1464.10-2007 [35], according to the requirements, the investigation included recording the occurrence of diseased mushrooms in each plot as well as calculating the incidence of *A. bisporus* and the control effect of fungicides. The *A. bisporus* harvest time was approximately 10 days after application, and the investigation results were obtained on 25 October 2021. The efficacy was calculated using the formula P = (CK − PT)/CK × 100%, where P represents the control effect percentage (%); CK represents the number of diseased mushrooms (fungi) in the blank control area; and PT represents the number of diseased mushrooms (fungi) in the treatment area. 

### 2.7. Effect of Fungicides on the Yield of A. bisporus

As for the effect of fungicides on the yield of *A. bisporus*, the fungicide test was conducted strictly in accordance with the requirements of the NY/T 1965.1-2010 [34]. The fungicide test was 1 time, 2 times, and 4 times the recommended dosage in the field, all at the active ingredient dosage. Spraying treatment took place when the mushroom buds rose from the covering soil, and the investigation was conducted after 12 days. The concentration of 1.8% seboctylamine acetate aqueous solution was set at 6, 12, and 24 mg/L, while the concentrations of prochloraz–manganese chloride complex wettable powder were set at 400, 800, and 1600 mg/L. A water treatment without chemical agents was used as the blank control. Each treatment was sprayed with 250 mL/m^2^ fungicide solution. The experiment design included random blocks for each plot, with each treatment repeated three times and an area of 2 m^2^. The five-point sampling method was used to collect 20 subentities in each cell.

### 2.8. Drug Residue Test

The drug residue test was conducted using QuECHERS to detect the residues of prochloraz–manganese salt and seboctylamine acetate in *A. bisporus* [36]. *A. bisporus* was planted in the mushroom shed of Shandong Agricultural University’s fungal resources and utilization training practice base. The test involved three treatments: 50% prochloraz–manganese chloride complex WP, active ingredient dosage, 400 mg/L; 1.8% seboctylamine acetate AS, active ingredient dosage, 6 mg/L; and a blank control. After 3 days of covering soil and fruiting, the test was divided. Each treatment also involved spraying 250 mL/m^2^ fungicide solution, and the experimental design was the same as that for determining the effect of fungicide on the yield of *A. bisprus*. Fresh *A. bisporus* samples were collected at 2 hours, 1 day, 3 day, 5 day, 7 day, and 9 day after application. From each plot, at least 1 kg of fresh *A. bisporus* was sampled by chopping. Two portions of 150 g samples were collected and stored at a low temperature in the refrigerator. The residual dynamics and final residues of prochloraz–manganese chloride complex and seboctylamine acetate in *A. bisporus* were calculated [25].

The QuECHERS method was used to prepare nutrient solutions and plant samples [36].

Chromatographic conditions: We used a poroshell 120 EC-C_18_; the chromatographic column temperature was 40 °C, with an injection volume of 5.0 μL and a flow rate of 0.8 mL/min. Mobile phase A was 0.1% formic acid in water, pH ≈ 3.5, while mobile phase B was acetonitrile.

The parameters of the mass spectrometer were as follows: The ion source was an Electrospray Ionization (ESI) source, with positive ion scanning mode and Multiple Reaction Monitoring (MRM) as the mass spectrometry scanning method. The ion source temperature (TEM) was set at 600 °C. The ion spray voltage (IS) was 4500 V. The curtain gas (CUR) flow rate was 30 psi, the nebulizer gas (Gas1) flow rate was 55 psi, the auxiliary gas (Gas2) flow rate was 60 psi, and the collision gas (CAD) pressure was 7 psi. The other mass spectrometry parameters are shown in Table 3.

### 2.9. Statistical Analysis

SPSS 25.0 software was used to calculate the biological activity regression equation, EC_50_ value, and 95% confidence limit of the agent against the mycelial growth of the pathogen of *A. bisporus*. A safety evaluation and a field efficacy test using Ducan’s method were performed to compare the differences between different treatments in each test. A value of *p* < 0.05 was considered statistically significant, and the data were expressed as mean ± standard deviation.

## 3. Results

### 3.1. Toxicity Measurement

**Figure 2:**
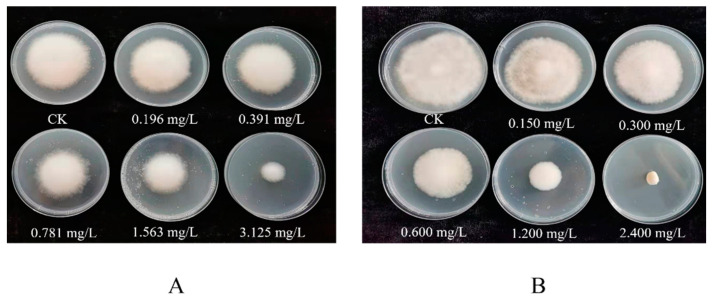
Bioassay testing of prochloraz–manganese chloride complex and seboctylamine acetate to *C. mycophilum*. (**A**) Seboctylamine acetate; (**B**) Prochloraz–manganese chloride complex.

To measure the inhibitory effects of nine fungicides on the mycelial growth of *C. mycophilum,* we used the mycelial growth rate method (Table 4). The EC_50_ values of quatrimycin, prochloraz–manganese chloride complex, seboctylamine acetate, prothioconazole, tebuconazole, difenoconazole, and polyoxin were 0.269, 0.939, 0.991, 0.240, 1.435, 6.589, and 4.396 mg/L, respectively. These fungicides exhibited high toxicity against the mycelial growth of cobweb disease in *A. bisporus*. Pyrimethanil and kresoxim-methyl had low toxicity against the mycelial growth of *A. bisporus*, with EC_50_ values of 34.584 and 31.271 mg/L, respectively (Figure 2). 

### 3.2. Spore Germination Test

The results of the inhibition of eight fungicides on the spore germination of *A. bisporus* are shown in Table 5. The EC_50_ values of these eight fungicides on spore germination range from 0.338 mg/L to 80.588 mg/L. Among them, seboctylamine acetate, tebuconazole, and quatrimycin showed strong inhibitory activity against spore germination; the EC_50_ values were 0.652, 0.338, and 1.365 mg/L, respectively. On the other hand, the inhibitory activities of prochloraz–manganese chloride complex, prothioconazole, pyrimethanil, difenoconazole, and kresoxim-methyl against the spore germination of *C. mycophilum* were found to be weak, with EC_50_ values of 80.588, 43.766, 13.393, 48.227, and 57.117 mg/L, respectively. These results indicate that both prochloraz–manganese chloride complex and seboctylamine acetate have good indoor activity against *C. mycophilum*.

The above results indicate that both prochloraz–manganese chloride complex and seboctylamine acetate exhibited effective inhibition against the mycelial growth and spore germination of the pathogenic fungus. 

### 3.3. Safety Test

**Figure 3:**
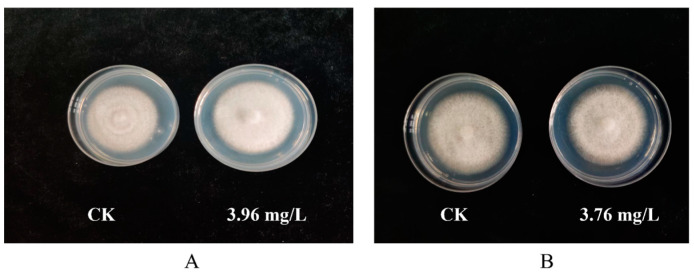
Safety of prochloraz–manganese chloride complex and seboctylamine acetate against *A. bisporus* mycelium. (**A**) Seboctylamine acetate; (**B**) prochloraz–manganese chloride complex.

Following the results of the indoor toxicity test, prochloraz–manganese chloride complex and seboctylamine acetate were chosen for further testing. The results of the indoor safety test results are presented in Table 6. The data indicate that at three different concentrations of 0.99, 1.98, and 3.96 mg/L of seboctylamine acetate and three concentrations of 0.94, 1.88, and 3.76 mg/L of prochloraz–manganese chloride complex, there were no significant differences in the inhibition of mycelial growth across the three varieties of *A. bisporus* (*A. bisporus* W2000, *A. bisporus* W192, and *A. bisporus* S2796 (Figure 3). This demonstrates that the use of these two agents on *A. bisporus* will not affect growth.

Based on indoor toxicity determination, field-recommended doses of prochloraz–manganese chloride complex and seboctylamine acetate were selected for follow-up tests. Table 7 clearly demonstrates that the application of prochloraz–manganese chloride complex at concentrations of 400 mg/L, 800 mg/L, and 1600 mg/L, as well as seboctylamine acetate at concentrations of 6 mg/L, 12 mg/L, and 24 mg/L, did not have any negative impact on the yield of *A. bisporus* fruiting bodies. Furthermore, there was no significant difference observed between the treated groups and the control group in terms of yield. Importantly, no signs of necrosis, deformity, or other adverse symptoms were noted, indicating that these two agents can be safely used on *A. bisporus*.

The indoor and field safety tests indicated that prochloraz–manganese chloride complex and seboctylamine acetate were not only highly toxic to *C. mycophilum*, but also safe for the growth of *A. bisporus*.

### 3.4. Control Effect of Prochloraz–Manganese Chloride Complex and Seboctylamine Acetate Against Cobweb Disease in A. bisporus

Table 8 summarizes the results of the efficacy test, revealing that both 50% prochloraz–manganese chloride complex WP and 1.8% seboctylamine acetate AS exhibited excellent control over the cobweb disease caused by *C. mycophilum* in *A. bisporus*. Spraying treatment was carried out when the mushroom buds appeared 10 days after soil covering. The test results after 10 days clearly indicate that 800 mg/L and 1600 mg/L concentrations of 50% prochloraz–manganese chloride complex WP were effective in controlling cobweb disease in *A. bisporus*, with control effects of 54.11% and 61.38%, respectively. It is evident that the higher the concentration, the better the control effect. Similarly, for 1.8% seboctylamine acetate AS, concentrations of 2 mg/L, 4 mg/L, and 6 mg/L were all found to be beneficial for preventing and treating cobweb disease in *A. bisporus*, with control effects of 71.76%, 78.82%, and 81.17%, respectively. Notably, the 6 mg/L concentration of 1.8% seboctylamine acetate aqueous solution achieved a control effect of 81.17%, suggested that this concentration offers the most optimal control among the tested doses.

### 3.5. Effects of Prochloraz–Manganese Chloride Complex and Seboctylamine Acetate Against Yield in A. bisporus

**Figure 4:**
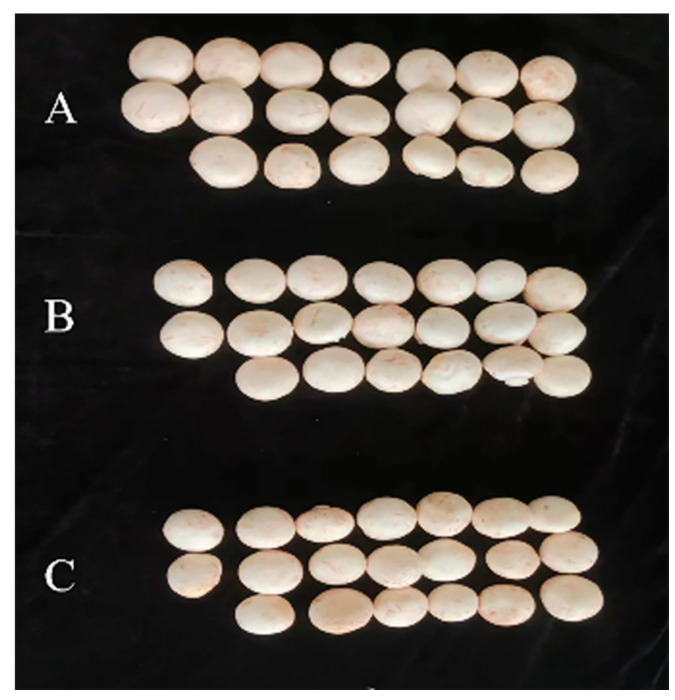
The effects of the highest concentrations of two fungicides on the yield of *A. bisporus.* (**A**) CK; (**B**) 24 mg/L 1.8% seboctylamine acetate; (**C**) 1600 mg/L 50% prochloraz–manganese chloride complex.

The results presented in Table 7 and Figure 4 indicate that the application of 50% prochloraz–manganese chloride complex WP and 1.8% seboctylamine acetate AS as chemical treatments did not significantly impact the yield of *A. bisporus* fruiting bodies compared to the untreated control group. There were no observed instances of necrosis or deformity in the mushrooms treated with these agents, further validating their safety for use on *A. bisporus*. Consequently, it can be confidently stated that 50% prochloraz–manganese chloride complex WP and 1.8% seboctylamine acetate AS are suitable for application in the management of cobweb disease in *A. bisporus* cultivation, ensuring both disease control and the preservation of mushroom quality and yield.

### 3.6. Effects of Prochloraz–Manganese Chloride Complex and Seboctylamine Acetate on Pesticide Residues in A. bisporus

**Figure 5:**
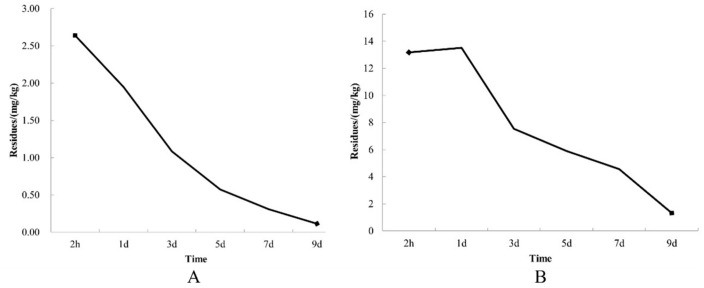
Degradation curves of chemical agents in *A. bisporus* fruiting body. (**A**) Degradation curve of seboctylamine acetate in sub-body of *A. bisporus* at 6 mg/L; (**B**) degradation curve of prochloraz–manganese chloride complex in fruiting body of *A. bisporus* at 400 mg/L.

The results regarding pesticide residue indicate that prochloraz–manganese chloride complex and seboctylamine acetate decompose relatively quickly. Specifically, when applied at concentrations of 1.8% for seboctylamine acetate aqueous solution (AS) and 50% for prochloraz–manganese chloride complex wettable powder (WP), with effective ingredient levels of 6 mg/L and 400 mg/L, respectively, the residual amounts after nine days of application at a rate of 250 mL per square meter were notably low. The final residue of seboctylamine acetate AS was measured at 0.116 mg/kg, while that of prochloraz–manganese chloride complex WP was 1.325 mg/kg. The maximum residue limit (MRL) for prochloraz–manganese chloride complex in edible fungi is 2 mg/kg, and our experimental results fell below this threshold, adhering to the national standard. For seboctylamine acetate, while a specific MRL for mushrooms is not established, the MRL for vegetables is 0.5 mg/kg. Notably, our experimental findings revealed residues of seboctylamine acetate to be less than 0.5 mg/kg, significantly lower than the established limit for vegetables (Figure 5). 

As a result, the application of 1.8% seboctylamine acetate AS and 50% prochloraz–manganese chloride complex WP during the fruiting bud stage of *A. bisporus* is deemed suitable. This practice effectively mitigates *A. bisporus* cobweb disease. The recommended dosages of active ingredients in the field are 1600 mg/L and 2–6 mg/L, respectively.

## 4. Discussion

As the *A. bisporus* planting industry rapidly develops, diseases affecting *A. bisporus* are becoming more common. Pathogens such as fungi, bacteria, and viruses pose a significant threat to *A. bisporus* production [9], with cobweb disease pathogens being especially prevalent. In China, cobweb disease in *A. bisporus* is caused by *C. mycophilum*, a pathogenic microorganism that infects mushrooms through their stomata, leading to the rapid decay and widespread contamination of surrounding mushrooms, resulting in substantial economic losses [11,37,38]. At present, chemical control measures are currently employed to manage *A.bisporus* cobweb disease, but the choice and number fungicides is limited, and there is a lack of field evaluation regarding the safety of these chemical agents [21]. Prochloraz-Mn and prochloraz have been utilized effectively to control mushroom diseases. Previous studies have demonstrated the inhibitory effects of prochloraz–manganese chloride complex and 2–benzo imidazole methyl carbamate on cobweb disease in edible mushroom [39], with Prochloraz–manganese chloride complex displaying effective antifungal properties. Additionally, research has shown the effectiveness of metrafenone (EC_50_ = 0.025 mg/L) and prochloraz-Mn (EC_50_ = 0.045 mg/L) as alternative fungicides for disease control [39]. Furthermore, prochloraz–manganese has been found to control green mold on white mushroom (*A. bisporus*) and increase the yield of *A. bisporus*, and can effectively control wet bubble (WBD) disease caused by *Mycogone rosea* while also increasing the yield of *A.bisporus* [19]. Chakwiya et al. focused on the sensitivity of *C. mycophilum* to carbendazim and prochloraz–manganese; prochloraz–manganese ED_50_ values varied from 0.00001 mg/L to 0.55 mg/L, and it is to be used in a disease management strategy [20]. Pathogen sensitivity to fungicides was estimated by probit analyses. Fungicide susceptibility tests showed that *C. mycophilum* strains were highly sensitive both to prochloraz (ED_50_ < 0.087 μg mL^−1^) and the newly introduced metrafenone (ED_50_ < 0.15 μg mL^−1^) [21]. Prochloraz can no longer be used in the European Union (EU) as of June 2023, and over-reliance on metrafenone has resulted in putative resistant pathogenic strains emerging. Prochloraz still showed good control of two different isolates of *C. mycophilum*, with efficacy values consistently reaching 70% [22]. This supports the findings of Stanojević et al. who also found that prochloraz performed better than the tested BCA in green mold and dry bubble disease trials in vivo [40]. However, the long-term use of prochloraz–manganese chloride complex can lead to resistance in mushroom disease, affecting its efficacy. For example, *C. mycophilum* type II isolate 192B1 has been found to develop resistance to prochloraz-Mn, rendering it ineffective in preventing cobweb disease symptoms [23]. In this study, we selected prochloraz–manganese chloride complex, due to its commonly used status in edible fungi, and found that it exhibited higher inhibitory toxicity towards the mycelial growth of *C. mycophilum*, and the EC_50_ was 0.939 mg/L. Discrepancies in the results between this study and others may be attributed to variations in pathogen virulence among different regions, which is also an important reason for this situation. In an effort to mitigate the development of resistance to a single fungicide, this study screened eight additional chemical fungicides, including seboctylamine acetate, a product currently under research and development in China. Seboctylamine acetate, a broad-spectrum fungicide with potential for controlling diseases in fruits and vegetables, demonstrated potent inhibitory effects against the mycelial growth and spore germination of *C. mycophilum*, with EC_50_ values of 0.990 mg/L and 0.652 mg/L, respectively. This finding presents a promising alternative for the prevention and treatment of *A. bisporus* cobweb disease in the future.

The application of fungicides for controlling mushroom crop diseases necessitates a thorough consideration of diverse factors, including the intricate interplay between edible fungi and pathogens. This interaction encompasses not only the modes of pathogen infection, but also the defensive mechanisms of mushrooms and sensitivity to fungicides. There is an urgent need for fungicides that have a good inhibitory effect on pathogens and are harmless to the host [41]. Hence, when employing chemical agents to combat pathogens, it is indispensable to assess their potential harm to *A. bisporus* strains, aiming to minimize or eliminate any adverse effects. Bhat et al. demonstrated that carbendazim is both highly efficacious and cost-effective in inhibiting the mycelial growth and conidial germination of pathogenic fungi, while posing no threat to the spores of edible fungi [42]. Liu et al. found that *Bacillus subtilis* B154 has antagonistic activity against *Alternaria alternata*, but does not affect the growth of *A. bisporus* [43]. Our results are similar to the results of Bhat et al. and Liu et al. [42,43] and show that the application of prochloraz–manganese chloride complex and seboctylamine acetate had no detrimental impact on the regular growth of *A. bisporus*. The field experiment results further revealed that at a concentration of 400 mg/L, prochloraz–manganese chloride complex achieved control efficacy of 61.38%. When the concentration of seboctylamine acetate was 6 mg/L, the control effect was 81.17%. The application of two chemical agents could prevent and control cobweb disease in *A. bisporus*, and does not affect the yield of *A. bisporus*.

Bioactive compounds extracted from plants or microorganisms can be used to prevent and treat diseases in edible fungi. These compounds have strong antifungal properties, especially in the treatment of spider web disease [41]. Clarke et al. also found that 48 h of *C. mycophilum* culture was exposed to 25% *v*/*v*, and 96 h of *B. velezensis* culture to a filtrate, and the biomass (*p* < 0.0002) was reduced by 57% [44]. Additionally, the essential oil extracted from cinnamon, geranium, and mint showed a good inhibitory effect on *C. mycophilum*, but caused damage to mushroom mycelium [17]. Furthermore, an EEO-TG emulsion prepared by Han et al. was able to inhibit a pathogen of *A. bisporus* from changing its membrane permeability [7]. However, the mechanism of action underlying biological pesticides is not well understood, potentially affecting their effectiveness compared to chemical fungicides in the field. Nevertheless, the burgeoning trend toward green agriculture presents a promising outlook for the development of biological pesticides in the realm of edible fungi [45].

Both chemical agents and biological pesticides are used to prevent and manage diseases, with a focus on minimizing adverse impacts on human health and the environment. It is crucial to safeguard crops from these diseases [41,46]. Pesticide residues can pose a significant risk to food safety, and the excessive use of pesticides during pest management can threaten human health [47]. Research has shown high occurrences of pesticide residues in edible fungi, with carbendazim, acephate, diuron, prochloraz, and dichlorvos being the predominant pesticides found. Wang et al. discovered that *Flammulina velutipes*, *Lentinus edodes*, and *Pleurotus ostreatus* exhibited a high occurrence of pesticide residues [38]. Chemical agents are commonly employed in the control of edible fungi. However, post-treatment, the residues that persist in agricultural products may exceed their permissible safe limits, thereby posing potential health hazards to humans [48]. To date, Han et al. have utilized an enhanced QuEChERS method coupled with ultrahigh-performance liquid chromatography–tandem mass spectrometry to accurately quantify xinjuan (seboctylamine) pesticide residues in plant-derived food products [49]. In addition, a method for the determination of seboctylamine acetate was established. The pesticide residue in *A. bisporus* is lower than the maximum limit standard of pesticide residue, so the modified agent is suitable for use in *A. bisporus*. In this study, a method for the determination of seboctylamine acetate residue was established, the results showed that the final residues of seboctylamine acetate and prochloraz–manganese chloride complex at 250 mL/m^2^ for 9 d were 0.116 and 1.325 mg/kg, respectively, which were lower than the maximum residue limit standard and could be used in *A. bisporus*. In a separate study that evaluated the residue levels of prochloraz–manganese chloride complex in grapes and soil, it was found that the minimum detection limit was 1.0 × 10^−10^ g, and the minimum detectable concentration was 0.04 mg/kg in grapes and 0.05 mg/kg in soil [50]. The findings presented in this study align with the aforementioned results, suggesting that the application of prochloraz–manganese chloride complex and seboctylamine acetate on *A. bisporus* is both safe and effective.

## 5. Conclusions

In conclusion, this study presented effective and safe fungicides to control cobweb disease in *A. bisporus*. It provides a detailed description of the residue determination analysis of the fungicide seboctylamine acetate in mushroom crop. This study proved that prochloraz–manganese chloride complex and seboctylamine acetate had a good inhibitory effect on the pathogen *C. mycophilum*. Moreover, the application of the two agents had no adverse effect on the mycelial growth and fruiting body growth of *A. bisporus*, and the residual amount of the agents was lower than the maximum residue limit standard. Field applications showed that 400 mg/L prochloraz–manganese chloride complex and 6 mg/L seboctylamine acetate gave 61.38% and 81.17% disease control, respectively.

## Figures and Tables

**Table 1:** Concentration gradient settings for 9 fungicides.

Fungicide	Experimental Chemical Concentration (mg/L)
Quatrimycin	0.075, 0.150, 0.300, 0.600, 1.200, 2.400, 4.800
Prochloraz–manganese chloride complex	0.150, 0.300, 0.600, 1.200, 2.400, 4.800, 9.600
Seboctylamine acetate	0.196, 0.391, 0.781, 1.563, 3.125, 6.250, 12.500
Prothioconazole	0.075, 0.150, 0.300, 0.600, 1.200, 2.400, 4.800
Tebuconazole	0.150, 0.300, 0.600, 1.200, 2.400, 4.800, 9.600
Difenoconazole	0.781, 1.563, 3.125, 6.250, 12.500, 25.000, 50.000
Pyrimethanil	6.250, 12.500, 25.000, 50.000, 100.000, 200.000
Kresoxim-methyl	6.250, 12.500, 25.000, 50.000, 100.000, 200.000
Polyoxin	0.781, 1.563, 3.125, 6.250, 12.500, 25.000, 50.000

**Table 2:** Concentration gradient settings for 8 fungicides.

Fungicide	Experimental Chemical Concentration (mg/L)
Quatrimycin	0.150, 0.300, 0.600, 1.200, 2.400, 4.800, 9.600
Prochloraz–manganese chloride complex	2.000, 4.000, 8.000, 16.000, 32.000, 64.000, 128.000
Seboctylamine acetate	0.075, 0.150, 0.300, 0.600, 1.200, 2.400, 4.800
Prothioconazole	2.000, 4.000, 8.000, 16.000, 32.000, 64.000, 128.000
Tebuconazole	0.150, 0.300, 0.600, 1.200, 2.400, 4.800, 9.600
Difenoconazole	2.000, 4.000, 8.000, 16.000, 32.000, 64.000, 128.000
Pyrimethanil	1.000, 2.000, 4.000, 8.000, 16.000, 32.000, 64.000
Kresoxim-methyl	2.000, 4.000, 8.000, 16.000, 32.000, 64.000, 128.000

**Table 3:** Residual test-related parameters.

Fungicides	Qualitative Ion Pair (*m*/*z*)	Quantitative Ion Pair (*m*/*z*)	DP (V)	CE (eV)	Retention Time (min)
Seboctylamine acetate	328.5/199.2	328.5/156.2	166	25/35	3.080
Prochloraz–manganese chloride complex	376.0/308.0	376.0/266.0	42	8/16	3.820

**Table 4:** Bioassay testing of 9 fungicides against *A. bisporus* cobweb disease.

Fungicides	Regression Equation	Coefficient of Determination	EC_50_/(mg·L^−1^)	95% Confidence Interval
Quatrimycin	y = 0.803 + 1.409x	0.927	0.269	0.095~0.582
Prochloraz–manganese chloride complex	y = 0.027 + 1.002x	0.932	0.939	0.171~3.093
Seboctylamine acetate	y = 0.006 + 1.645x	0.968	0.991	0.367~2.696
Prothioconazole	y = 0.820 + 1.324x	0.809	0.240	0.001~0.574
Tebuconazole	y = −0.150 + 0.956x	0.864	1.435	0.426~12.328
Difenoconazole	y = −0.585 + 0.714x	0.892	6.589	-
Pyrimethanil	y = −2.077 + 1.350x	0.663	34.584	15.469~324.450
Kresoxim-methyl	y = −1.843 + 1.233x	0.773	31.271	13.563~340.752
Polyoxin	y = −1.044 + 1.623x	0.623	4.396	1.129~19.953

**Table 5:** Toxicity of 8 fungicides against spores of *C. mycophilum*.

Fungicides	Regression Equation	Coefficient of Determination	EC_50_/(mg·L^−1^)	95% Confidence Interval
Prochloraz–manganese chloride complex	y = −1.958 + 1.027x	0.677	80.588	55.482~125.775
Seboctylamine acetate	y = 0.264 + 1.417x	0.845	0.652	0.509~0.824
Prothioconazole	y = −3.636 + 2.216x	0.38	43.766	24.923~91.635
Pyrimethanil	y = −1.564 + 1.388x	0.506	13.393	7.598~21.968
Difenoconazole	y = −2.025 + 1.203x	0.824	48.227	32.280~85.313
Kresoxim-methyl	y = −3.191 + 1.816x	0.5	57.117	31.357~276.730
Tebuconazole	y = −1.346 + 2.86x	0.912	0.338	0.295~0.387
Quatrimycin	y = −1.563 + 1.970x	0.933	1.365	0.897~3.652

**Table 6:** Indoor safety test of prochloraz–manganese chloride complex and seboctylamine acetate on *A. bisporus*.

Fungicides	Effective Concentration/(mg·L^−1^)	*A. bisporus* W2000		*A. bisporus* W192		*A. bisporus* S2796	
		Colony Diameter/(cm)	Inhibition Ratio	Colony Diameter /(cm)	Inhibition Ratio	Colony Diameter/(cm)	Inhibition Ratio
CK	-	6.43 ± 0.17 a	-	6.52 ± 0.18 a	-	6.51 ± 0.16 a	-
Prochloraz–manganese chloride complex	0.94	6.28 ± 0.05 ab	0.02	6.41 ± 0.06 a	0.02	6.23 ± 0.05 bcd	0.04
	1.88	6.25 ± 0.03 ab	0.03	6.35 ± 0.06 a	0.03	6.17 ± 0.06 cd	0.05
	3.76	6.13 ± 0.04 b	0.05	6.33 ± 0.06 a	0.03	6.14 ± 0.06 d	0.06
Seboctylamine acetate	0.99	6.32 ± 0.05 ab	0.02	6.39 ± 0.07 a	0.02	6.39 ± 0.05 abcd	0.02
	1.98	6.26 ± 0.07 ab	0.03	6.37 ± 0.07 a	0.02	6.26 ± 0.10 abcd	0.04
	3.96	6.22 ± 0.07 ab	0.03	6.29 ± 0.10 a	0.04	6.25 ± 0.09 abcd	0.04

Note: Different lowercase letters in the same column in the table indicate significant differences at the 0.05 level between different treatments.

**Table 7:** Field safety test of prochloraz–manganese chloride complex and seboctylamine acetate on *A. bisporus* (effect on yield).

Fungicides	Effective Concentration/(mg·L^−1^)	Yield/(g)
CK	-	529.3 ± 21.7 a
Prochloraz–manganese chloride complex	400	519.9 ± 3.4 a
	800	502.1 ± 10.4 a
	1600	492.6 ± 3.6 a
Seboctylamine acetate	6	493.0 ± 3.9 a
	12	527.5 ± 10.0 a
	24	530.0 ± 16.6 a

Note: Yield is the fresh weight of *A. bisporus* per 20 fruited bodies of different treatments (unit: g). Different lowercase letters in the same column in the table indicate significant differences at the 0.05 level between different treatments.

**Table 8:** Field efficacy test of prochloraz–manganese chloride complex and seboctylamine acetate on cobweb disease in *A. bisporus*.

Fungicides	Experimental Chemical Concentration/(mg·L^−1^)	Incidence/(%)	Control Effect/(%)
CK	-	61.0	-
Prochloraz–manganese chloride complex	400	29.4	49.41 ± 6.35 d
	800	26.6	54.11 ± 5.69 d
	1600	22.4	61.38 ± 4.63 c
Seboctylamine acetate	2	16.7	71.76 ± 3.65 b
	4	12.4	78.82 ± 2.89 a
	6	10.9	81.17 ± 6.51 a

Note: Different lowercase letters in the same column in the table indicate significant differences at the 0.05 level between different treatments.

## Data Availability

The data that support the findings of this study will be available from the corresponding author upon reasonable request.
